# Selective Laser Sintering of Laser Printed Ag Nanoparticle Micropatterns at High Repetition Rates

**DOI:** 10.3390/ma11112142

**Published:** 2018-10-31

**Authors:** Filimon Zacharatos, Ioannis Theodorakos, Panagiotis Karvounis, Simon Tuohy, Nuno Braz, Semyon Melamed, Ayala Kabla, Fernando de la Vega, Kostas Andritsos, Antonios Hatziapostolou, Dimitris Karnakis, Ioanna Zergioti

**Affiliations:** 1 Physics Department, Zografou Campus, National Technical University of Athens, 15780 Athens, Greece; jtheod@mail.ntua.gr (I.T.); kostas_andritsos13@hotmail.com (K.A.); zergioti@central.ntua.gr (I.Z.); 2 School of Engineering, University of West Attica, Campus 1, 12243 Aigaleo, Greece; panos.karvounis12@gmail.com (P.K.); ahatzi@teiath.gr (A.H.); 3 Oxford Lasers Ltd., 8 Moorbrook Park, Didcot, Oxon OX11 7HP, UK; simon.tuohy@oxfordlasers.com (S.T.); nuno.braz@oxfordlasers.com (N.B.); Dimitris.Karnakis@oxfordlasers.com (D.K.); 4 PV Nano Cell Ltd., 8 Hamasger st., P.O. Box 236 Migdal Ha’Emek, Migdal Haemek 2310102, Israel; simon@pvnanocell.com (S.M.); ayala@pvnanocell.com (A.K.); fernando@pvnanocell.com (F.d.l.V.)

**Keywords:** laser sintering, laser induced forward transfer, silver nanoparticle inks, heat affected zone, high speed laser processing

## Abstract

The increasing development of flexible and printed electronics has fueled substantial advancements in selective laser sintering, which has been attracting interest over the past decade. Laser sintering of metal nanoparticle dispersions in particular (from low viscous inks to high viscous pastes) offers significant advantages with respect to more conventional thermal sintering or curing techniques. Apart from the obvious lateral selectivity, the use of short-pulsed and high repetition rate lasers minimizes the heat affected zone and offers unparalleled control over a digital process, enabling the processing of stacked and pre-structured layers on very sensitive polymeric substrates. In this work, the authors have conducted a systematic investigation of the laser sintering of micro-patterns comprising Ag nanoparticle high viscous inks: The effect of laser pulse width within the range of 20–200 nanoseconds (ns), a regime which many commercially available, high repetition rate lasers operate in, has been thoroughly investigated experimentally in order to define the optimal processing parameters for the fabrication of highly conductive Ag patterns on polymeric substrates. The in-depth temperature profiles resulting from the effect of laser pulses of varying pulse widths have been calculated using a numerical model relying on the finite element method, which has been fed with physical parameters extracted from optical and structural characterization. Electrical characterization of the resulting sintered micro-patterns has been benchmarked against the calculated temperature profiles, so that the resistivity can be associated with the maximal temperature value. This quantitative correlation offers the possibility to predict the optimal process window in future laser sintering experiments. The reported computational and experimental findings will foster the wider adoption of laser micro-sintering technology for laboratory and industrial use.

## 1. Introduction

The increasing advancement of flexible/stretchable and large area electronics has broadened the spectrum of the applications which benefit from their unique characteristics: Flexible and stretchable sensors [1], low cost and low weight non pervasive wearable systems [2], optically transparent tactile devices [3], are a few examples of the application potential offered by this rapidly evolving field. At the same time, the increasingly demanding requirements of such applications have highlighted the technological value of digital and high resolution additive manufacturing processes. Among a plethora of additive manufacturing technologies, laser based additive processing, i.e., laser printing and laser sintering, stands out owing to the unique attributes it offers: Processing of materials in liquid and solid phase, high resolution, and minimal damage to the printed or sintered material and underlying substrate. For highly viscous dispersions, e.g., pastes, with high metal nanoparticle content, laser printing is particularly interesting as it has no restriction in terms of viscosity: In this work we will demonstrate the high speed and reproducible printing of Ag ink micropatterns with metal content up to 75% and viscosity higher than 15,000 cP. Post-printing laser sintering can be efficiently employed for the selective transformation of the printed patterns to solid tracks with electrical behavior comparable to bulk metals. Pioneering studies in laser sintering of nanoparticle dispersions have been published over 15 years ago [4], already demonstrating <10× bulk resistivity. Numerous papers have reported on inkjet printed metal nanoparticle micropatterns which showed high conductivity after post-printing laser sintering. In particular, in References [5,6] the authors report on laser sintered, inkjet printed patterns comprising Ag nanoparticles, with resistivity down to 5× bulk using 60 s irradiation of continuous wave laser on glass substrates. In Reference [7], the authors fabricated air stable OFETs using inkjet printing of Au nanoparticle inks and low temperature selective laser sintering, with a carrier mobility of 0.002 cm^2^/Vs and a I_on_/I_off_ ratio ranging from 10^3^ to 10^4^. Other selective sintering methods have been reported over the past decade, e.g., Öhlund et al. have shown that inkjet printed Ag nanoparticles with an average size of <10 nm can be efficiently sintered using electrical and microwave sintering as well as photonic curing, with resistivity values down to <2× bulk Ag [8]. The aforementioned low temperature sintering techniques show significant promise for specific case studies, however present serious limitations with respect to laser sintering: Photonic curing using flash lamps exposes the whole surface of the samples with maximized lateral heat affected zone; electrical sintering although selective and efficient, is a serial and slow process, with low throughput; microwave sintering [9] presents high selectivity but is a time consuming and nanoparticle size dependent method. Other low temperature sintering techniques involve chemical sintering by organic solvent dipping [10] and plasma sintering [11]. The former offers no lateral selectivity, while a special case of the latter has been demonstrated in Reference [12], with lateral resolution around 200 μm and resulting resistivity <10× bulk Ag for a sintering duration of a few minutes. Selective laser sintering stands out owing to the micrometer scale lateral and in depth resolution, fast processing time up to 10 m/s, which are further pronounced when it is combined with laser printing using the same configuration. Over the past decade, laser sintering has been established as a reliable and minimally invasive process, compatible with additive manufacturing of electronic devices [13], sensors [14], circuit bonding and integration [15]. Numerous studies have proposed different laser sources: UV, visible and Infra-Red (IR) [16] lasers, continuous wave [17,18] up to ultra-short femtosecond (fs) pulsed lasers [19], as the most appropriate sintering solution for a variety of nanostructured materials i.e., Ag [20] and Cu [21,22] nanoparticles, Ag nanowires [23] and carbon nanotubes [24], ceramics [25]. In this direction, the authors have previously conducted a comparative study between the efficiency of continuous wave (CW), nanosecond (ns) and picosecond (ps) pulsed laser sources, concluding that ns pulses at 532 nm can sinter efficiently Ag nanoparticle ink micropatterns, over a large process window [26]. This finding was further supported in Reference [27], in which the authors applied the same process for the fabrication of high performance flexible RF transmission lines and in Reference [28] for printed electrodes for flexible sensors. However, many challenges need to be further addressed before exploiting the full potential of laser sintering at the micrometer scale: The laser pulse width effect on laser sintering is not yet fully clarified, in particular for pulse width variation of one order of magnitude from 10–100 ns, a regime which many commercially available and affordable laser systems operate in. The industrial requirements for high speed processing, dictate high repetition rates. While the laser process window has been determined in terms of laser power or laser fluence for low speed processing with CW or pulsed lasers [29], for high speed processing at high repetition rates, i.e., >~100 kHz, sintering is dependent mostly on the average laser power and the pulse to pulse overlap [30,31], in which case the standardization of laser process parameters is challenging. Moreover, the online measurement of temperature variations using pyrometry and CCD camera imaging is currently unattainable at the nanosecond time scale [32], therefore such methods are of value for long pulse laser processing, or for post-processing determination of the temperature of the irradiated area [33]. Although several previous works have simulated the effect of single laser pulses on the temperature distribution of the irradiated interface [6,34], in effect, pulsed laser sintering is accomplished as an accumulative result of a large number of pulses. In this work, we have simulated the accumulative effect of high repetition rate pulses at 90% overlap in order to build a realistic computational model which can predict the maximal superficial temperature for any given set of laser parameters (wavelength, pulse width, pulse energy, repetition rate, pulse to pulse overlap, etc.). The same model can simulate the in-depth temperature profile for the ink layer depending on the substrate optical and thermal properties. Temperature dependent material properties have been fed into the model after corresponding structural and thermal characterization. The selected laser process parameters are based on a substantial number of experimental laser sintering iterations for varying pulse width, ranging between 20 and 200 ns, a regime compatible with low cost laser systems currently commercially available. The corresponding repetition rate was 60–450 kHz and average laser power 180–2000 mW, which have resulted in high quality laser sintering of laser printed linear patterns of sub 100 μm width, with scanning speeds as high as 1 m/s and resistivity down to <6× bulk Ag. The resulting laser sintering process window is applicable on the laser printed linear structures, with high yield (95%) and reproducibility, despite the complex geometry of these micropatterns. Four point probe electrical measurements of the resulting sintered micro-patterns has been benchmarked against the calculated temperature profiles, so that the resulting resistivity can be associated with the simulated temperature value. The simulated temperatures have been further validated by oven sintering experiments, at the corresponding temperatures and the resulting measured resistivities are in good qualitative agreement with the simulated ones. The benchmarking of the simulated versus the experimental results has substantial value for quantitative prediction of the resulting temperature: This quantitative correlation offers the possibility to calculate the expected temperature distribution for high repetition rate ns laser sintering, and hence the optimal process for the aforementioned applications. The resulting micro-patterns have been characterized structurally and morphologically, so as to verify that uniform in-depth sintering across each micro-pattern’s surface has been actually accomplished. The reported computational and experimental findings will foster the wider adoption of laser micro-sintering technology for laboratory and industrial use.

## 2. Materials and Methods

**Figure 1:**
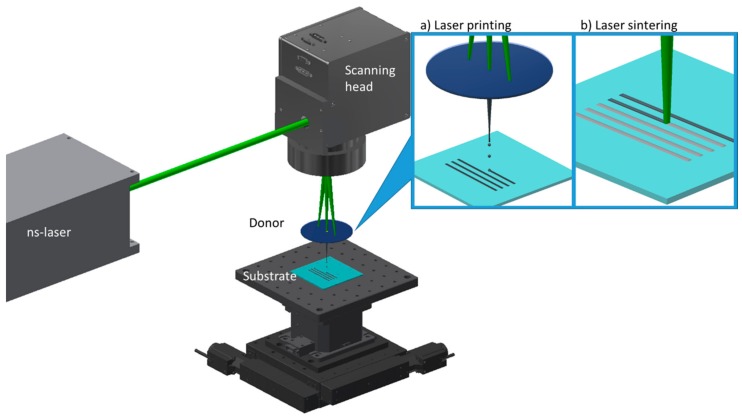
High speed laser printing and laser sintering set-up schematic representation.

The Ag nanoparticle highly viscous inks involved in this study were developed by P.V. Nano Cell Ltd. (Migdal Ha’Emek, Israel), and have solid content in the range 70–75% wt, with a resulting viscosity which typically ranges between 15,000–100,000 cP at shear rate 1 s^−1^. Quartz donor substrates and PolyethyleneNaphthalate (PEN) Teonex^®^ 125 μm thick films, which were used as receiving substrates, are also commercially available products. PEN’s glass transition temperature is 121 °C and melting point is at 269 °C, which make this substrate an excellent candidate for the investigation of thermal damage during laser sintering. A two-fold process combining laser printing and laser sintering has been developed the purposes of the reported work, using the same laser experimental set-up. The high speed printing process was implemented by the Laser Induced Forward Transfer method without any sacrificial layer as described in Reference [35], but with several key-modifications in terms of laser source, scanning system and printable material, which enable high speed and reproducibility. The post printing process consisting of high speed laser sintering using the same experimental set-up is the main focus of this study. The experimental configuration of the two-fold process, comprised a nanosecond pulsed laser source at 532 nm, with variable pulse width in the range of 20–200 ns, varying as a function of the repetition rate (see Supplementary Information Figure S1), which can go up to 500 kHz, and has max output power 20 W, a 2D packaged galvanometer scan head with max scan speed 5 m/s and sub-micron step resolution, combined with motorized x-y-z CNC stages for the donor-substrate relative positioning (Figure 1). 

For the electrical characterization of laser sintered micropatterns, a four point probe IV station implementing the Van Der Pauw method was utilized.

A numerical model has been designed and developed specifically for optimizing the laser sintering process, using the ANSYS Mechanical finite element software (ANSYS Inc., Canonsburg, PA, USA). In particular the three dimensional (3D) heat transfer at the interface of an Ag ink layer and a PEN substrate has been investigated, in order to calculate the superficial and the in-depth temperature distributions after the effect of a single or multiple laser pulses. Temperature distribution of the Ag layer and PEN are equally important as the former relates to the efficiency of the sintering and the latter determines the heat affected zone on the substrate. The optical and thermal properties of the involved materials have been considered with the following assumptions:

For the Ag ink, solid material with optical absorption coefficient measured at 532 nm at room temperature has been fed into the model. UV-Vis reflection and transmission spectroscopy from 350 to 1100 nm was employed for the calculation of the optical absorption coefficient of Ink layer: A silver ink with metal content 70%, which was the less viscous ink involved in this study, was spin coated at 8000 rpm for 5 min. The resulting transmission and reflection spectra can be found in Supplementary Information Figure S2.

From these spectra the absorbance of the ink versus a broad range of wavelengths was calculated, as were other interesting properties, such as the optical absorption coefficient, extinction coefficient and optical penetration depth. The following Table 1 synopsizes the aforementioned properties for selected wavelengths which could be of interest for laser sintering. Differential Scanning Calorimetry (DSC) measurements were carried out in order to define the heat capacity, (Cp), of the high viscous Ag ink up to 200 °C, which is shown in Supplementary Information, Figure S2c and has been fed into the model. For 200–400 °C extrapolated values have been considered. Measured mass density for the silver ink layer after drying is 7 g/cm^3^ owing to air voids which are formed in the interior of the layer during drying. For the thermal conductivity (k(T)) temperature dependence has been considered according to the literature [36]. A transient thermal analysis computational model was built using finite element analysis. Our goal is the accurate simulation of the in-depth temperature distribution between Ag-Ink and PEN.

The initial temperature was set at 22 °C (room temperature). Total number of time steps was set at 160–200 on average, while timestep size varied from 1 ns to 100 ns.

**Figure 2:**
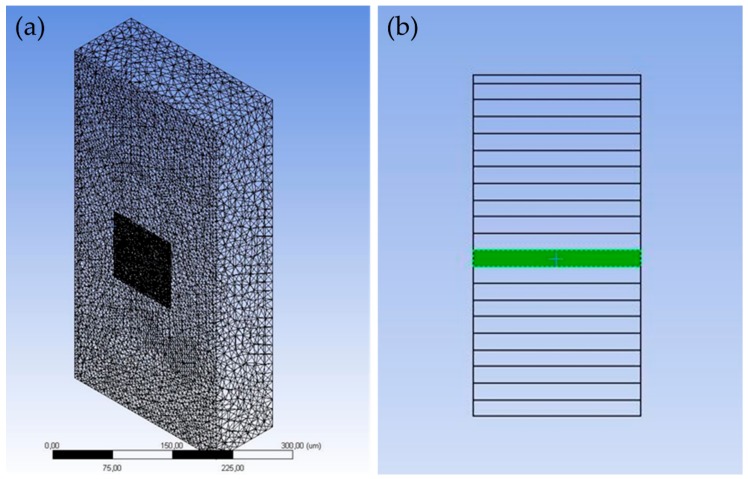
(**a**) Mesh characteristics of the two building blocks of the model. The Ag-ink plane has denser meshing (dark grey cuboid), and the PEN plane has a lot sparser meshing (light grey cuboid); (**b**) For the 10 consecutive pulses case, the selected area which has received heat flux from 10 pulses is highlighted in green.

The simulated laser wavelength was 532 nm and the laser intensity in space was modeled by a Gaussian function. The thermal effect of laser pulse irradiation can be described by the 3D heat equation with a source term to represent absorption of light. Heat flux was applied at the Ag ink plane considering only heat conduction, and the optical penetration depth was considered according to Beer lambert’s law, as in a different case study comprising Ag nanowires by the authors, employing the same simulation scheme [37]. The temporal distribution of the pulse is considered top hat as presented for an e.g., 30 ns pulse width. From 0 to 1 ns the heat flux increases gradually to reach maximum at the beginning of the 1st ns. At the end of the 30th ns the heat flux decreases in the same way in order to reach 0 W/m^2^ at the 31st ns. The mesh characteristics are presented in Figure 2 and Table 2: The Orthogonal Average Quality is an important measure of the mesh quality and varies from 0 to 1, with the latter being the best possible quality. In our case this value is approximately 0.79, indicating an adequate mesh quality for avoiding discretization errors. Grid sensitivity analysis was performed in order to determine the optimal grid in terms of accuracy and time efficiency for our case study.

## 3. Results and Discussion

### 3.1. Experimental Results and Characterization

#### 3.1.1. High Speed Laser Printing

**Figure 3:**
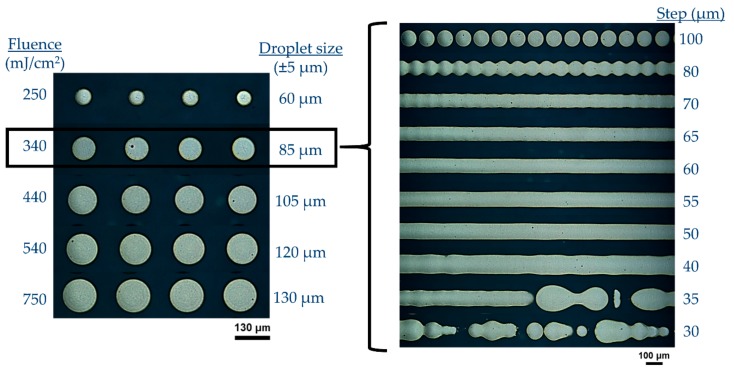
Experimental optimization of the printing parameters for achieving uniform and long lines with sub-100 μm width comprising highly viscous Ag nanoinks.

Arrays of 10 mm long linear micropatterns with width in the order of 80–90 μm and submicron thickness were printed on PEN substrates by developing and optimizing a high speed laser printing process with no sacrificial layer. Parameters of interest are donor receiver gap, laser fluence, repetition rate, and scanning speed which define the pulse to pulse distance. After numerous sets of experiments, the optimized parameters used in this study were: Wavelength 532 nm, fluence ~300 mJ/cm^2^, spot to spot distance (step) 55 μm (Figure 3). For the purposes of oven sintering experiments (see following paragraphs), similar printing conditions were applied for the formation of linear micropatterns on glass substrates.

#### 3.1.2. Oven Sintering

**Figure 4:**
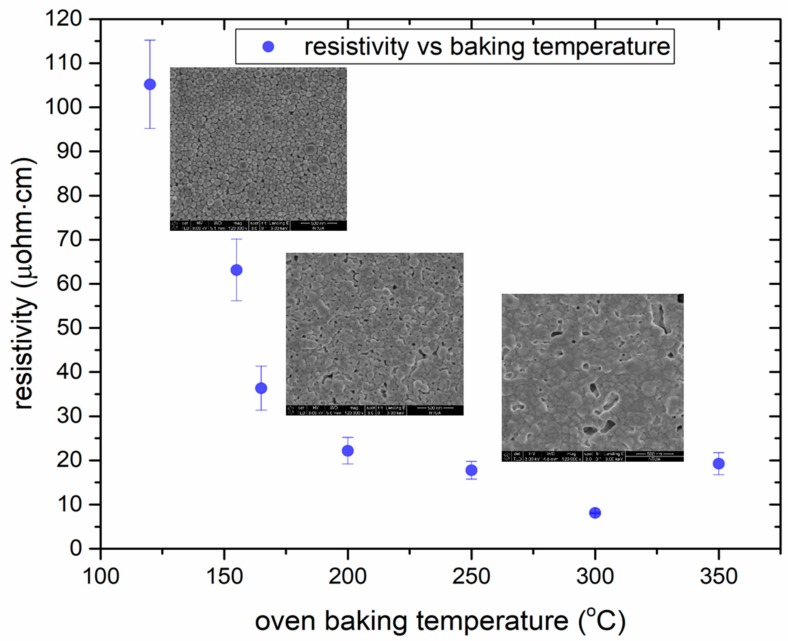
Resistivity as a function of oven baking temperature, results obtained from sintering in an oven for 4 h. Error bars correspond to deviation from 10 different samples measured for each temperature. Morphology of the ink layer and the grain size increase is presented in three different SEM micrographs corresponding to (from left to right): 150, 200 and 300 °C. In the latter temperature, sintering has been fully accomplished and this reflected by both the grain morphology and the low resistivity (~10 μOhm·cm).

Laser printed structures on glass substrates have been oven baked at temperatures exceeding the glass transition temperature point of PEN, ranging from 125–350 °C, in order to investigate the efficiency of sintering carried out by a thermal process, prior to laser sintering. In this case only the electrical properties of the resulting sintered structures are of interest, whereas the adhesion or the thermal damage induced to the substrate are not part of this study. Figure 4 synopsizes the resulting resistivity values measured from 10 different sets of samples baked at the aforementioned temperatures for 4 h. Below 250 °C the sintering is not optimal in terms of resulting resistivity. Judging also from the resulting morphology, it can be seen from the corresponding SEM pictures that for 250 °C for 4 h, grain size is smaller and necking is less pronounced with respect to the 300 °C case and that the minimum resistivity is achieved for 300 °C as, for higher temperatures the resistivity increases. Oven sintering was conducted in ambient air, as was laser sintering for direct comparison. Ag is not substantially oxidized during baking at temperatures <300 °C, since the oxidation process has a very slow rate. For higher temperatures, oxidation may be significant [38], and this is why the oven baking process was conducted up to 350 °C, and not in higher temperatures. Although these values cannot be directly associated with the laser sintering results, they can serve as a quantitative benchmark, which can add further value to the modelling results presented in the next section (Section 3.2).

#### 3.1.3. High Speed Laser Sintering: Process Development

**Figure 5:**
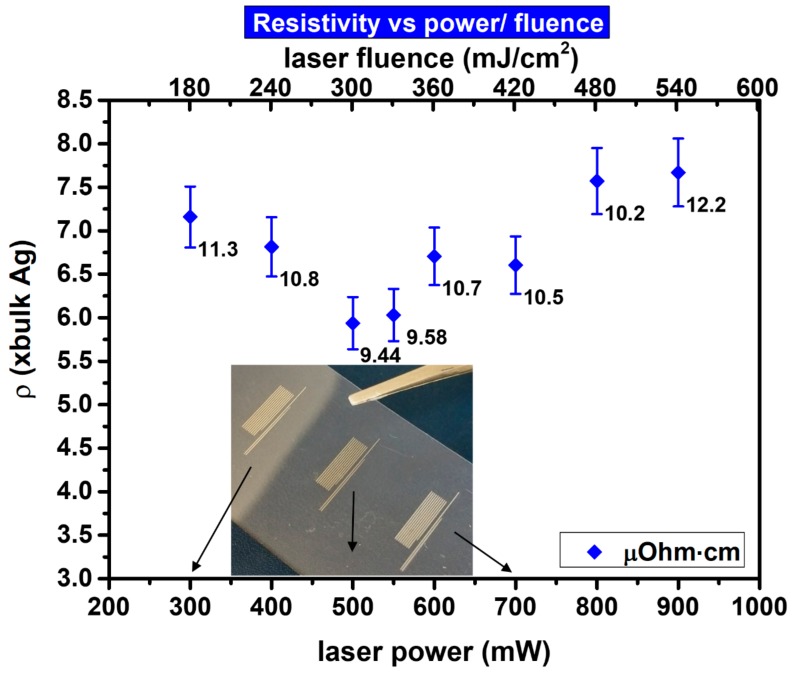
Laser power/fluence vs. resistivity for 98% pulse to pulse overlap. Sintering is accomplished for a wide range of laser power without severe thermal damage on the PEN substrate.

A high speed laser sintering process capable for achieving <10× bulk Ag resistivity on laser printed linear micropatterns has been developed in the frame of this study. Laser printed structures were left to dry in ambient atmosphere for 60 min and then exposed to laser sintering at 532 nm with high pulse to pulse overlap (>90%) and scanning speed >100 mm/s. In as deposited inks, dispersing agents separate the nanoparticles from each other, preventing interaction between particles. As the heating process begins, the solvent as well as other volatile additives must be evaporated first (Figure 4 left SEM image) before the actual sintering can occur. In this case, the solvent and the volatile additives of the ink, were evaporated in room temperature, be leaving the ink to dry for 1 h. Indeed, this could be facilitated by other sintering methods, such as hot plate baking at low temperature (e.g., 60 °C), or low power cw laser sintering at near-IR wavelength (e.g., 800 nm). However, this step would complicate the process and would add extra components to the configuration. The concept of this work was to demonstrate the efficiency and yield of utilizing exactly the same experimental set-up for laser sintering of laser printed micropatterns, using low cost commercially available high repetition rate laser sources. When laser irradiation begins, heating induced by the impinging photons lead to necking which causes the particles to agglomerate (Figure 4 middle SEM image). In (Figure 4 right SEM image) the particles have sintered, forming a conductive pattern. The nanoparticle heating at this stage facilitates the evaporation of the remaining additives. The morphology of the sintered structure and the resulting surface roughness and pores is defined by numerous factors including the maximal temperature achieved, the laser power and pulse width, the evaporation rate of the solvent’s additives and the shape of necking. Initial experiments with the aforementioned experimental set-up and Ag highly viscous ink showed significant promise and high reproducibility (<50× times bulk resistivity on >90% of irradiated patterns). Significant effort was invested on improving the resistivity by varying laser power only, before investigating the effect of other parameters. The best result achieved in this study in terms of resistivity was 9.44 ± 0.5 μOhm·cm ({5.93 ± 0.3} × bulk Ag) for 500 mW laser power, 60 kHz repetition rate, 100 mm/s scanning speed and 20 ns pulse width. The following graph (Figure 5) shows the experimental parameters varied in order to obtain this. A total of 10 different measurements to different lines sintered under the same conditions provided the experimental data used in this and all the following graphs. The developed process enables the reproducible (yield > 90%) sintering of laser printed linear structures with length:width aspect ratio 100:1. 

**Figure 6:**
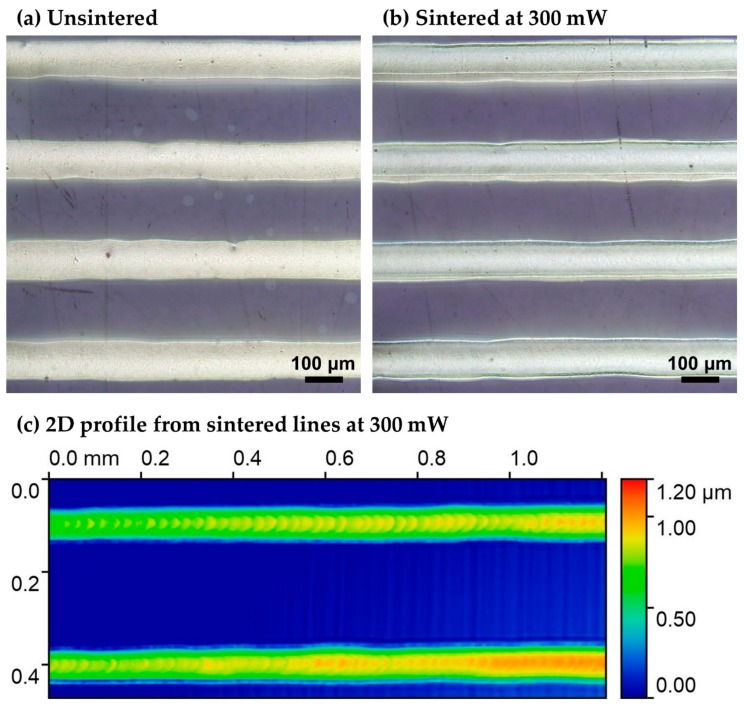
Optical microscope scans for: (**a**) Laser printed, non-sintered lines; (**b**) Laser printed and laser sintered lines with optimal conditions and (**c**) Color chart of height profile of the two bottom sintered lines presented in (**b**).

The following Figure 6 shows the morphology of the laser printed Ag micropatterns prior to and after laser sintering using a 3D profiler (Sensofar S lynx, Sensofar Metrology Spain). Although it is usual to observe shrinkage of a pattern containing nanoparticles after laser sintering, in this study, the high metal content of the Ag ink ensures that there is no significant variation in the thickness of the lines. The thickness of the unsintered patterns is 1.2 μm and as can be seen from the following 3D profiler images, the post sintering thickness decrease is in the range of 10–20% (see Figure 6c).

**Figure 7:**
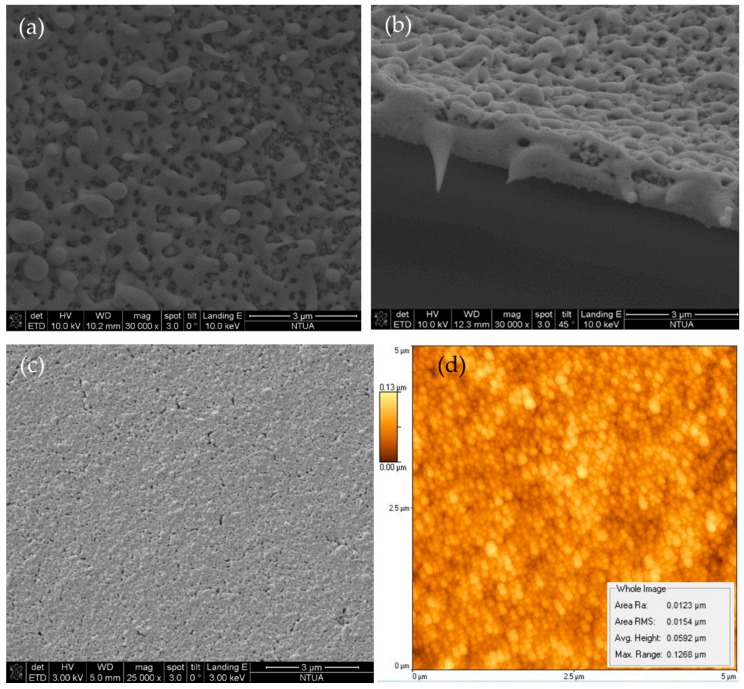
SEM micrographs of laser sintered line: (**a**) Top view; (**b**) Side view of cleaved structure; (**c**) Top view of laser sintered Ag surface morphology for optimized sintering conditions and (**d**) AFM micrograph indicating average RMS roughness and max height measured of sample (**c**).

In order to confirm that laser sintering has been accomplished from top to bottom and not only at the superficial layers of the micropatterns, side view SEM imaging has been employed. The following Figure 7b reveals that when laser sintering is successful, Ag NPs are sintered from top to bottom across the whole thickness of the structure. This set of pictures has been selected because it clearly shows the similarity in morphology of the surface and on the side-wall of the cleaved structure, proving that sintering has been achieved across the whole thickness of the micropattern. Although the surface roughness is in the order of 200 nm in this case, after optimizing the laser sintering process parameters, one can achieve surface roughness <100 nm, as can be seen in Figure 7c. However, the average RMS roughness is significantly smaller in both cases. According to the Atomic Force Microscope (AFM) image in Figure 7d, the rms roughness does not exceed 20 nm, while the maximal height difference is in the order of 100 nm.

#### 3.1.4. High Speed Laser Sintering: Pulse Width Effect Investigation

In this section we report on the investigation of the effect of laser power under constant pulse width. As soon as we had concluded on the optimal power in terms of resistivity, we started scanning the pulse width for this specific power and some other power values around the optimum (constant power study). Next, we performed the pulse width study for constant pulse energy and we present the best results which were obtained for 6 μJ pulse energy. That way, we could clarify the contribution of the pulse width under either constant power or constant pulse energy conditions. 

**Figure 8:**
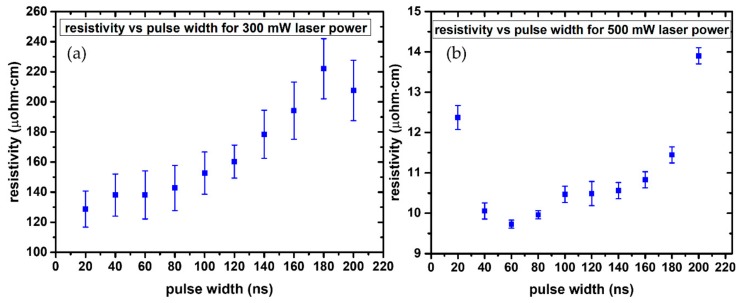
Typical diagram of Resistivity vs. laser pulse width for constant incident laser power of 300 mW (**a**) and 500 mW (**b**) for 10 different pulse widths. Error bars reflect the standard deviation from 10 different measurements.

The contribution of laser pulse width to the sintering efficiency on flexible substrates, within a range of picosecond to nanosecond pulse widths, has been highlighted in a previous study [26], concluding that nanosecond pulses provide the optimal results. In order to shed more light on the nanosecond regime, a major focus of this work is the experimental investigation of the effect of laser pulse width within a refined range of 20–200 ns, to define the optimal processing parameters for the fabrication of highly conductive Ag patterns at high laser repetition rates on PEN substrates. Two different sets of experiments were conducted to this end: In both cases the repetition rate increased for increasing pulse width, and in order to maintain a fixed overlap between two consecutive laser pulses, the scanning speed of the laser beam had to increase as well. In the first case, the experiments were conducted at constant laser power (in the range of 180–1000 mW), while in the second case constant laser pulse energy was employed (in the range of 1–10 μJ). For the case of constant laser power (e.g., 300 mW), typical results indicate a systematic increase of resistivity for increasing pulse width, as can be observed from Figure 8a. Having already optimized the sintering condition in terms of resulting resistivity, for a constant power of 500 mW, we selected this value for reproducing the constant power experiment. The results are presented in Figure 8b. In this case, since the resistivity values are overall lower than in the case of 300 mW, and in particular for pulse widths 40–80 ns the standard deviation becomes significant and as a result resistivity is fluctuating around 10 μOhm·cm. For 20 ns, the pulse duration is shorter than the optimal duration, as will also be discussed in Section 3.2.

The following Table 3 contains the experimental parameters involved in this study. From the contents of this table, it is made clear that as the pulse width increases with increasing repetition rate, the pulse energy (and consequently the fluence) decreases. This partially accounts for this systematic increase of resistivity, although in high speed laser processing, laser power, which is kept constant throughout this experiment, is also a very important factor.

**Figure 9:**
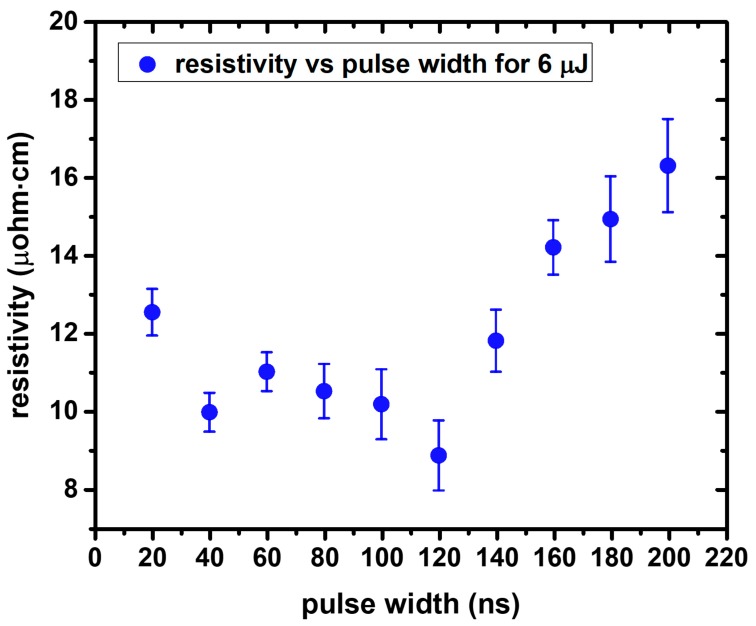
Resistivity (ρ) as a function of pulse width for constant pulse energy. With increasing repetition rate, average power increases as well. The minimal ρ value in this experiment corresponds to 120 ns pulse width and 1500 mW power. Higher average power values are detrimental and induce structural damage to the sintered structure, resulting in higher resistivity.

The results from the constant power experiment gave rise to the need for a constant pulse energy study (Figure 9). An intermediate value between 5 and 8.33 μJ was selected for this experiment, 6 μJ in particular. Higher pulse energies induced very high temperatures which were detrimental to the substrate, whereas lower pulse energies resulted in insufficient sintering.

Although the lower ρ value is, in this case, 8.91 ± 0.9 μOhm·cm, the deviation is higher than in the case of the previous experiment, therefore the value 9.44 ± 0.5 μOhm·cm is considered as the optimal achieved in this study. Furthermore, in the next paragraph we will clarify why it is for the interest of the process to keep the laser power at the lowest possible levels.

**Figure 10:**
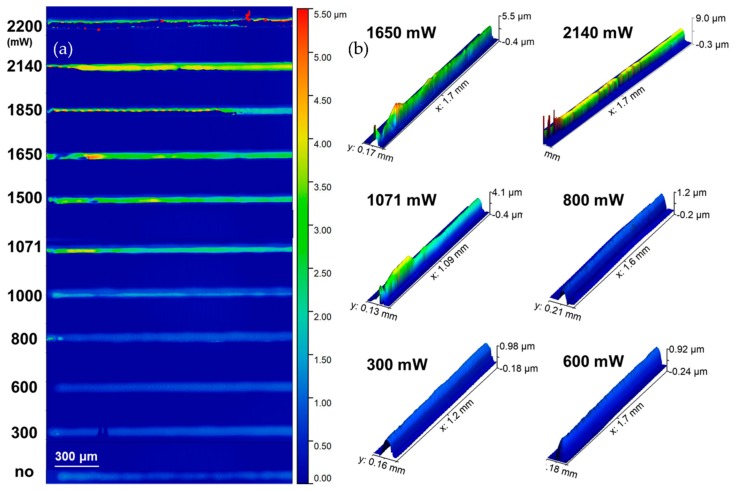
3D optical microscope micrographs: (**a**) 11 lines scanned for increasing power from 300–2200 mW; (**b**) selected 3D scans indicating that in low powers, sintering results in decreased line height, whereas for high laser power, thermal effects induce damage to both the substrate and the sintered structure itself, causing severe deformations and spikes.

The effect of the increasing laser power on the morphology of the sintered lines is highlighted in Figure 10, for lines sintered under constant pulse energy according to the parameters contained in Table 4. As a general remark, one can observe that for low laser powers (<800 nm), the effect of laser sintering is, as expected, the shrinkage of the height of the lines due to the removal of solid additives. However, as the power increases, (>800 mW) so do first, the degradation of the substrate, which swells and deforms the sintered line’s geometry and second, the actual line itself demonstrates severe indications of delamination, spikes and roughness. The bottom line is that although sintering might be accomplished even in high laser powers, it is always desirable to achieve low resistivity at low powers for the sake of the involved materials and energy saving.

### 3.2. Modelling

In this work we employed the 3D computational model described in Section 2, which solved the heat transfer equation at the Ag ink/PEN interface using the commercial software of ANSYS Mechanical, so as to calculate the in depth temperature distribution across this interface after the effect of a single laser pulse. For the appropriate scale of the solution, a solver specifically designed for micrometer-scale sized problems (μmKS solver) was employed and room temperature was set at 293 K. When a short laser pulse is focused on the surface, it is assumed that the incident photons are absorbed instantaneously to a penetration depth, l_z_, which is inversely proportional to the optical absorption coefficient α(T).

The amount of energy absorbed is described by the Beer-Lambert’s law. The heating effect of a single laser pulse is well described by the three dimensional heat equation given by (1):

(1) $$Q\left(x,y,z,t\right)=\rho \left(T\right)\mathrm{Cp}\left(T\right)\frac{\partial T\left(x,y,z,t\right)}{\partial t}-\nabla \cdot \left(k\left(T\right)\nabla T\left(x,y,z,t\right)\right),$$

**Figure 11:**
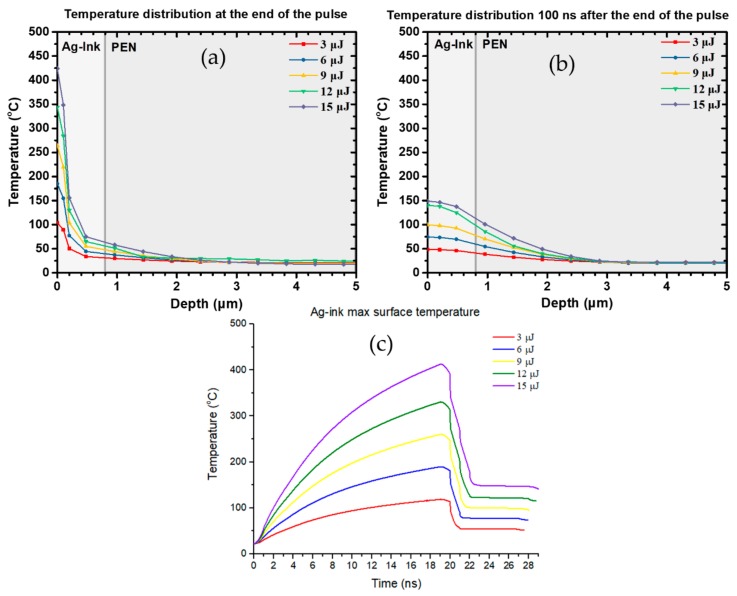
The in-depth profile of temperature distribution following the incidence of a 20 ns pulse has been calculated for pulse energy 3, 6, 9, 12 and 15 μJ: (**a**) At the end of the pulse, the superficial temperature varies substantially for varying pulse energy; (**b**) A total of 100 ns after the effect of the pulse, the temperature increase induced in the interior of PEN is maximized. Still, the heat affected zone on PEN is very restricted regardless of the pulse energy and (**c**) As expected, the superficial temperature reaches the maximal value at the end of 20 ns.

The spatial and temporal variation of the temperature and material properties are on the right part of this equation. ρ(Τ) defines the mass density of the material while Cp(T) and k(T) are the thermal properties that can be found on Table 1 for PEN and Ag ink. The material properties which were fed into the model are, for the case of PEN, the ones provided by the manufacturer, whereas for the Ag ink, measurements have provided the temperature dependent parameters, as described in Section 2. Heat conduction has only been considered. By varying laser parameters such as laser pulse energy, pulse width, repetition rate and total number of incident laser pulses, one can determine the optimal process conditions for achieving uniform sintering without significant damage on the PEN substrate. Furthermore, by benchmarking electrical characterization of the resulting sintered micro-patterns against the calculated temperature profiles, the conductivity can be associated with the maximal temperature value. This quantitative correlation offers the possibility to predict the optimal process window in future laser sintering experiments and define the optimal process window while maintaining PEN’s heat affected zone restricted to a few microns in depth. Focusing first on single laser pulses, we investigated the effect of increasing pulse energy: In Figure 11, the calculated in-depth temperature distributions are shown immediately after a single incident pulse for five (5) different pulse energies ranging from 3–15 μJ at 532 nm, for 20 ns pulse width. The maximal superficial temperature achieved for each pulse energy is depicted in Figure 11c, and it occurs at the end of the pulse duration. It is very interesting however, to know how the in-depth temperature profile is shaped as a function of time. By investigating several cases, we can claim that for the first tens of nanoseconds after the effect of the pulse, the superficial temperature of the Ag ink is dropping, while the temperature at the Ag ink/PEN interface is rising, as heat transfer occurs from the Ag ink layer’s surface to the interior. At a certain point, i.e., 100 ns after the effect of the pulse for 20 ns pulse width, the temperature at the Ag ink/PEN interface reaches the maximal value, as can be seen from Figure 11b, but for all these simulated pulse energy values, remains well below 121 °C which is the PEN glass transition temperature, and the in depth heat affected zone of PEN is less than 2 μm thick. Therefore, despite the sufficiently high temperatures (>250 °C) reached at the Ag ink surface for pulse energies >9 μJ, the maximal temperatures achieved at the other end of the Ag ink layer are significantly lower. This would potentially lead to inhomogeneous or partial sintering in an experimental process. Moreover, this is one of the main reasons why single pulse laser sintering at low repetition rates might require higher pulse energy than when high repetition rate lasers are employed, which would incur more severe damage to the underlying PEN substrate. In all cases, Ag layer thickness is 800 nm and the effective area, corresponding to laser spot size, is 100 μm × 100 μm.

**Figure 12:**
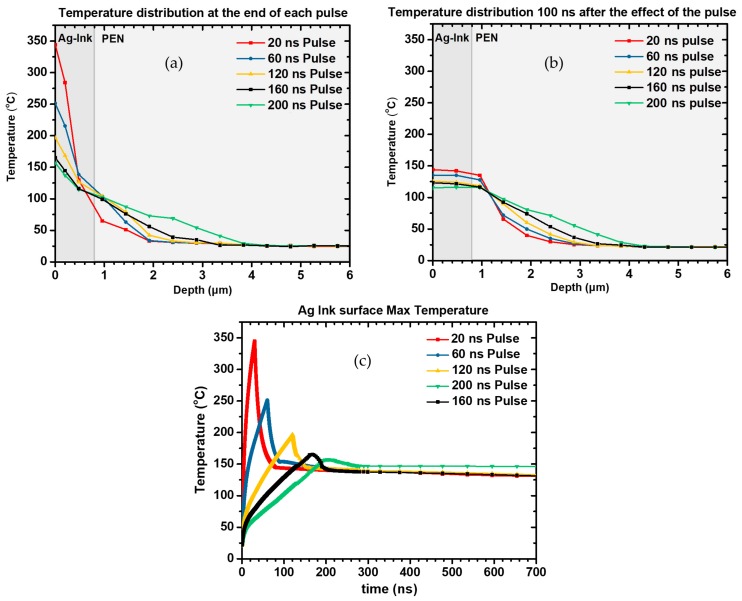
In-depth profile of temperature distribution at the end of each pulse has been calculated for pulse width 20, 60, 120, 160 and 200 ns. The superficial temperature varies substantially for varying pulse width (**a**); The heat affected zone on PEN becomes significant for 200 ns, 100 ns after the incidence of the pulse (**b**) and The maximal temperature achieved on the Ag ink layer decreases for increasing pulse width, as the peak power decreases as well (**c**).

The main focus of the single laser pulse simulation was the clarification of the effect of the pulse width on the resulting temperature profile. In Figure 12, the calculated temperature distributions for five (5) different pulse widths immediately after the pulse incidence for fixed pulse energy 12 μJ, are presented. It is evident that the longer the pulse width, the lower the superficial temperature immediately after the heat flux has ceased. On the contrary, the heat diffusion is higher for longer pulse widths. A total of 100 ns after the effect of each pulse the temperature profile has dramatically changed, and the huge difference in temperature at the surface has now converged to a mean temperature of about 130 °C ranging from 142 °C for 20 ns pulse to 118 °C for 200 ns. These calculated profiles refer to single shot effects and cannot be directly associated with experimental results obtained from the laser pulse width variation study conducted for high repetition rate scanning. Nevertheless, they can provide very useful indications about how heat flows for the outer surface of the Ag ink layer to the interior, and how heat is transferred near the Ag/PEN interface, by calculating the corresponding temperature profiles.

**Figure 13:**
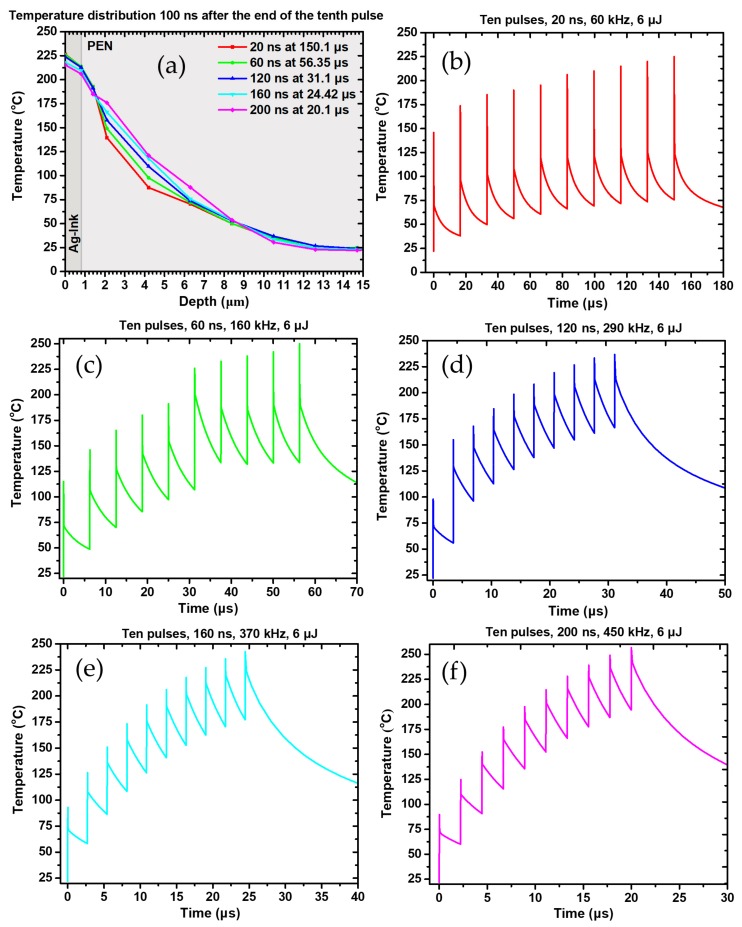
(**a**) Temperature distribution 100 ns after the end of the pulse for five different pulse widths. Simulated max temperature profiles after the effect of 10 pulses for 6 μJ pulse energy: (**b**) 20 ns; (**c**) 60 ns; (**d**) 120 ns; (**e**) 160 ns and (**f**) 200 ns pulse width.

The highlight of this numerical study is the investigation of the accumulative thermal effect of 10 pulses on the interface of interest. All the experiments described in Section 3.1 have been conducted at high repetition rates (>60 kHz) and high pulse to pulse overlap (>90%), which result in corresponding speed >100 mm/s for a spot size of 100 μm. The 10-pulse numerical study is intended to simulate the actual experimental process conditions and, in this respect, the aforementioned parameters have been fed into the model. For this specific, 10-pulse study, multiple irradiated areas have been considered, which were formed by 90% overlapping squares of 100 μm side. After solving the heat transfer equation, the overall irradiated was sliced in distinct regions of 10 μm × 100 μm, and the 10th slice has been selected for the analysis of the temperature distribution. This specific slice has received heat flux by 10 consecutive wide pulses of 6 μJ. The repetition rate is set at 60 KHz, so the overall simulated process has a duration of several μs. Figure 13 clearly demonstrates that consecutive pulses have an accumulative effect on the temperature profile, which reaches a plateau at the 9th pulse. Between the 1st and the 9th pulse there is more than a 40% temperature increase. For the multi pulse case, as we can observe from Figure 13, the temperature increase becomes significant after the 5th pulse. In this temperature regime, grain size increase may significantly affect Ag thermal conductivity, *k*, according to Reference [39], but the calculations carried out in Reference [5] indicate that for laser fluences commensurate with our experiment, the resulting thermal conductivity is a fraction of the bulk Ag’s one. Therefore, in this specific multi pulse case study, this grain size dependence of *k* has been omitted, as not of major contribution, in order to save computational time and develop a functional computational model.

Three main conclusions can be drawn from these graphs: First, it is evident that for repetition rates 120 kHz the maximum temperature achieved is similar and does not exceed 250 °C in any case. This can be attributed to the fact that, despite the gradual increase of average power, the peak power of each pulse decreases with increasing pulse width. This is also reflected by the max temperature achieved after the effect of the first pulse, which is getting smaller as the repetition rate increases. As the repetition rate increases and the duty cycle of the laser becomes shorter, but the pulse duration increases. Therefore, the cooling time between each pulse is getting shorter and the lowest calculated temperature during each cycle is getting higher. It is evident, that for a large number of pulses, the average temperature of the irradiated area during laser sintering will be a lot higher for high repetition rates (i.e., >120 kHz) with respect to smaller repetition rate, and that is expected to facilitate a more uniform and efficient sintering. In the next section, a comparison of the calculated and the experimental laser and oven sintering results is presented.

### 3.3. Benchmark of Numerical vs. Experimental Results

The oven sintering experiments conducted for this study allow us to directly associate the sintering temperature with the resulting resistivity. Although there are some fluctuations among 10 different samples and, in particular, around 300 °C, all the data converge to a minimum in terms of resistivity for this specific temperature (ρ < 10 μOhm·cm). For lower, as well as for higher average temperatures, the resistivity values are always higher, but quite close to the aforementioned value for 250 °C (see Figure 4). It is safe to consider therefore, that it is desirable to locally and temporarily reach and exceed 250 °C at the Ag ink plane during laser sintering. On top of that, laser sintering results are at least comparable and sometimes better than the oven sintering ones in terms of resistivity (see Figure 5). The numerical calculations conducted in this study confirm that for a wide range of pulse energies (6–15 μJ) and pulse widths (20–200 ns) it is feasible to achieve temperatures higher than 300 °C even for single shot irradiation. For the particular case of 6 μJ, a simulation calculating the effect ten consecutive pulses on the temperature profiles of the Ag ink layer has been conducted (Figure 13), and confirms that temperatures around 250 °C are typically achieved, with increasing average temperature as the repetition rate increases. An experimental case study for 6 μJ pulse energy (Figure 9) validates the findings of the corresponding numerical study, demonstrating that the lowest ρ value in this experiment corresponds to 120 ns pulse width and 1500 mW power (8.91 ± 0.9 μOhm·cm). However, for long pulse widths, high average power values are detrimental to the sintering process and induce structural damage to the sintered structure, resulting in higher resistivity, so it is for the interest of the process to keep the laser power at the lowest possible levels, therefore the value of 9.44 ± 0.5 μOhm·cm for 500 mW power is preferable. The validated simulated temperatures can be quantitatively associated with the experimental results for the case of constant power (Figure 8): For 300 mW power the temperatures achieved are always lower than 250 °C while for the case of 500 mW, the average temperatures achieved are around or higher than 300 °C.

### 3.4. Performance Evaluation—Adhesion Testing

**Figure 14:**
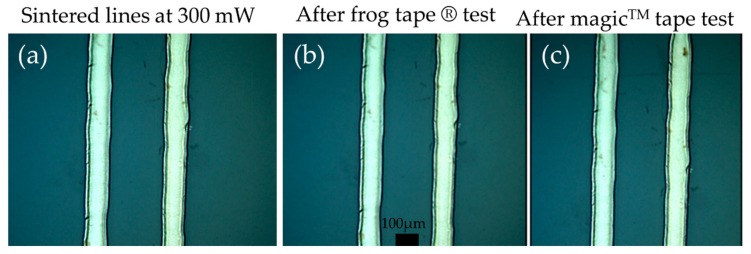
Microscope photos before and after the adhesion testing: (**a**) sintered lines; (**b**) the same lines after frog tape^®^ and (**c**) magic^TM^ tape tests.

In order to evaluate the adhesion of the laser printed and laser sintered micropatterns, a set of peeling tests using two types of tapes was conducted. Arrays of non-sintered lines and sintered lines at 300, 500, and 700 mW with the conditions described in the process development section. In this direction the test samples have undergone Frog Tape^®^ Delicate Painting Tape adhesion testing, (180° Peel Adhesion to Stainless Steel: 1.1 N/cm from product manufacturer’s datasheet) and Scotch No. 810 Magic™ Tape testing (180° Peel Adhesion to Stainless Steel: 2.5 N/cm). All the sintered structures have survived both tests without any observable cracking or defects (see Figure 14) and no measurable change in resistance. However, the non-sintered patterns exhibited minor and severe cracking after frog tape and delicate tape respectively. Therefore, it can be safely concluded that, during laser sintering, the adhesion of the structures to PEN improves significantly, owing to the laser induced thermal load, which while otherwise undesirable, it can be employed to the benefit of the process in this particular case.

## 4. Conclusions

In this work we have successfully employed high repetition rate laser processing for the selective sintering of laser printed micropatterns comprising Ag nanoparticles, with resulting resistivity down to <6× bulk. We have demonstrated for the first time in literature, that despite the complex non ideal geometry of the laser printed patterns, laser sintering can be effectively applied over a wide process window, which has been clarified by numerous experimental and computational results. By conducting simulation for varying pulse width with multiple laser pulses, the former show that for longer pulse widths the heat affected zone increases, and the latter have shown that the resulting temperature values increase for increasing repetition rate. Furthermore, we have focused on the effect of the laser pulse width on the resulting sintering efficiency, as pulse widths in the 10–100 ns regime can be found in many commercially available laser systems interesting for industrial applications. By varying either laser power or laser pulse energy we have concluded that over a wide range of parameters, <10 μOhm·cm resistivity can be achieved: For 20 ns pulse width, the optimal power in terms of ρ is 500 mW and for this power the most appealing pulse width is 60 ns. For constant pulse energy of 6 μJ the most appealing pulse widths are 40 and 120 ns, corresponding to 600 and 1500 mW average power. Overall, a wide range of average laser powers results in low resistivity for pulse widths 20–120 ns, however post sintering morphological and structural characterization reveal that the structural damage and number of cracks and spikes increases with increasing laser power. One should target at achieving the lowest possible resistivity by keeping the laser power to the minimum. The benchmarking of the numerical versus the experimental results indicates that average powers around 500 mW are sufficient in order to obtain temperatures over 300 °C, which are necessary for the efficient sintering of the Ag nanoparticles involved in this study.

## Figures and Tables

**Table 1:** Involved materials’ properties.

Property	Ag Ink	PEN
α(Τ) (cm^−1^) at 32 nm	1.20 × 10^5^	negligible
k (W/mK)	140	0.15
Cp (J/kgK)	**	1300
ρ (g/cm^3^)	7	1.38
R (–)	0.5	negligible

** Can be found in Supplementary Information.

**Table 2:** Mesh characteristics.

Elements	44,561
Nodes	72,827
Size Function	Adaptive
Conforming Method	Tetrahedrons
Orthogonal Average Quality	0.79

**Table 3:** Experimental conditions employed for the pulse width study under constant power.

Pulse Width (ns)	Rep. Rate (kHz)	Scan Speed (mm/s)	Average Power	Laser Spot Diameter (FWHM) μm	Estimated Laser Pulse Energy (μJ)		Estimated Laser Fluence (mJ/cm^2^)	
20	60	100	300/500	100	5/	8.33	168/	280
40	120	200			2.5/	4.16	84/	140
60	160	266			1.87/	3.12	63/	105
80	200	333			1.5/	2.50	50/	83
100	250	416			1.4/	2.00	40/	67
120	290	483			1/	1.66	33/	55
140	330	550			0.91/	1.52	31/	51
160	370	616			0.81/	1.35	27/	45
180	430	716			0.7/	1.17	24/	40
200	450	750			0.66/	1.10	22/	37

**Table 4:** Experimental conditions employed for the pulse width study under constant pulse energy.

Pulse Width (ns)	Rep. Rate (kHz)	Scan Speed (mm/s)	Pulse Energy (μJ)	Laser Spot Diameter (FWHM) (μm)	Measured Laser Power (mW)
20	60	100	6	100	300
40	120	200			600
60	160	266			800
80	200	333			1000
100	250	416			1071
120	290	483			1500
140	330	550			1650
160	370	616			1850
180	430	716			2140
200	450	750			2200

## Supplementary Materials

The following are available online at http://www.mdpi.com/1996-1944/11/11/2142/s1, Figure S1: Performance of the high repetition rate laser utilized in this study according to the manufacturer. Pulse width increases for increasing repetition frequency, Figure S2: Measured T (a) and R (b) spectra for 180 nm thick spin coated Ag nanoparticle ink for the 350–1100 nm range. (c) Cp vs. T for the same ink.

## References

[1] Bao Z., Chen X. (2016). Flexible and Stretchable Devices. Adv. Mater..

[2] Liu Y., Pharr M., Salvatore G.A. (2017). Lab-on-Skin: A Review of Flexible and Stretchable Electronics for Wearable Health Monitoring. ACS Nano.

[3] Lee J., Lee P., Lee H., Lee D., Lee S.S., Ko S.H. (2012). Very long Ag nanowire synthesis and its application in a highly transparent, conductive and flexible metal electrode touch panel. Nanoscale.

[4] Bieri N.R., Chung J., Haferl S.E., Poulikakos D., Grigoropoulos C.P. (2003). Microstructuring by printing and laser curing of nanoparticle solutions. Appl. Phys. Lett..

[5] Lee D.G., Kim D.K., Moon Y.J., Moon S.-J. (2013). Estimation of the Properties of Silver Nanoparticle Ink During Laser Sintering via In-Situ Electrical Resistance Measurement. J. Nanosci. Nanotechnol..

[6] Lee D.G., Kim D.K., Moon Y.J., Moon S.-J. (2013). Effect of laser-induced temperature field on the characteristics of laser-sintered silver nanoparticle ink. Nanotechnology.

[7] Ko S.H., Pan H., Grigoropoulos C.P., Luscombe C.K., Fréchet J.M., Poulikakos D. (2007). All-inkjet-printed flexible electronics fabrication on a polymer substrate by low-temperature high-resolution selective laser sintering of metal nanoparticles. Nanotechnology.

[8] Öhlund T., Örtegren J., Andersson H., Nilsson H.-E. Sintering methods for metal nanoparticle inks on flexible substrates. Proceedings of the International Conference on Digital Printing Technologies.

[9] Perelaer J., Gans B.J., Schubert U.S. (2006). Ink-jet printing and microwave sintering of conductive silver tracks. Adv. Mater..

[10] Wakuda D., Hatamura M., Suganuma K. (2007). Novel method for room temperature sintering of Ag nanoparticle paste in air. Chem. Phys. Lett..

[11] Reinhold I., Hendriks C.E., Eckardt R., Kranenburg J.M., Perelaer J., Baumann R.R., Schubert U.S. (2009). Argon plasma sintering of inkjet printed silver tracks on polymer substrates. J. Mater. Chem..

[12] Wunscher S., Stumpf S., Teichler A., Pabst O., Perelaer J., Beckert E., Schubert U.S. (2012). Localized atmospheric plasma sintering of inkjet printed silver nanoparticles. J. Mater. Chem..

[13] Ko S.H., Chung J., Pan H., Grigoropoulos C.P., Poulikakos D. (2007). Fabrication of multilayer passive and active electric components on polymer using inkjet printing and low temperature laser processing. Sens. Actuators A.

[14] Zhu B.L., Xie C.S., Wang A.H., Wu J., Wu R., Liu J. (2007). Laser sintering ZnO thick films for gas sensor application. J. Mater. Sci..

[15] Liu W., Wang C., Wang C., Jiang X., Huang X. (2017). Laser Sintering of Nano-Ag Particle Paste for High-Temperature Electronics Assembly. IEEE Trans. Compon. Packag. Manuf. Technol..

[16] Paeng D., Yeo J., Lee D., Moon S.J., Grigoropoulos C.P. (2011). Laser wavelength effect on laser induced photo-thermal sintering of silver nanoparticles. Adv. Mater..

[17] Auyeung R.C.Y., Kim H., Mathews S.A., Piqué A. (2007). Laser Direct-Write of Metallic Nanoparticle Inks. J. Laser Micro Nanoeng..

[18] Ermak O., Zenou M., Toker G.B., Ankri J., Diamand Y.S., Kotler Z. (2016). Rapid laser sintering of metal nanoparticles inks. Nanotechnology.

[19] Son Y., Yeo J., Moon H., Lim T.W., Hong S., Nam K.H., Yoo S., Grigoropoulos C.P., Yang D.Y., Ko S.H. (2011). Nanoscale Electronics: Digital Fabrication by Direct Femtosecond Laser Processing of Metal Nanoparticles. Adv. Mater..

[20] Peng P., Hu A., Zhou Y. (2012). Laser sintering of silver nanoparticle thin films: Microstructure and optical properties. Appl. Phys. A.

[21] Halonen E., Heinonen E., Mäntysalo M. (2013). The Effect of Conditions Laser Sintering Process Parameters on Cu Nanoparticle Ink in Room Conditions. Opt. Photonics J..

[22] Zenou M., Ermak O., Saar A., Kotler Z. (2014). Laser sintering of copper nanoparticles. J. Phys. D Appl. Phys..

[23] Spechler J.A., Arnold C.B. (2012). Direct-write pulsed laser processed silver nanowire networks for transparent conducting electrodes. Appl. Phys. A.

[24] Gao Y., Liang F., Freihofer G., Wu B., Mohan B., Raghavan S., Gou J., Li S., Albee B., Bishnoi S.W. (2011). Laser sintering of carbon nanotube-reinforced ceramic nanocomposites. Int. J. Smart Nano Mater..

[25] Basile N., Gonona M., Petit F., Cambier F. (2012). Interaction between laser beam and BaTiO3 powders in selective laser sintering treatments. J. Eur. Ceram. Soc..

[26] Theodorakos I., Zacharatos F., Geremia R., Karnakis D., Zergioti I. (2015). Selective laser sintering of Ag NPs ink for applications in flexible electronics. Appl. Surf. Sci..

[27] Zacharatos F., Iliadis N., Kanakis J., Bakopoulos P., Avramopoulos H., Zergioti I. (2016). Laser Direct Writing of 40 GHz RF Components on Flexible Substrates. J. Opt. Laser Technol..

[28] Zacharatos F., Makrygianni M., Geremia R., Biver E., Karnakis D., Leyder S., Puerto P., Delaporte P., Zergioti I. (2016). Laser Direct Write micro-fabrication of large area electronics on flexible substrates. Appl. Surf. Sci..

[29] Myungo J., Byoungyoon L., Sooncheol J., Myeongkyu L. (2012). Comparative studies on thermal and laser sintering for highly conductive Cu films printable on plastic substrate. Thin Solid Films.

[30] Kumpulainen T., Pekkanen J., Valkama J., Laakso J., Tuokko R., Mantysalo M. (2011). Low temperature nanoparticle sintering with continuous wave and pulse lasers. Opt. Laser Technol..

[31] Lu C.T., Lu F.L., Tsai C.E., Huang W.H., Liu C.W. (2017). Process Simulation of Pulsed Laser Annealing on Epitaxial Ge on Si. ECS J. Solid State Sci. Technol..

[32] Chivel Y., Smurov I. (2010). On-line temperature monitoring in selective laser sintering/melting. Phys. Procedia.

[33] Berumen S., Bechmann F., Lindner D., Kruth J.-P., Craeghs T. (2010). Quality control of laser- and powder bed-based Additive Manufacturing (AM) technologies. Phys. Procedia.

[34] Cheng C.W., Chen J.K. (2016). Femtosecond laser sintering of copper nanoparticles. Appl. Phys. A.

[35] Makrygianni M., Kalpyris I., Boutopoulos C., Zergioti I. (2014). Laser induced forward transfer of Ag nanoparticles ink deposition and characterization. Appl. Surf. Sci..

[36] Lide D.R. (1998). Chemical Rubber Company Handbook of Chemistry and Physics.

[37] Zacharatos F., Karvounis P., Theodorakos I., Hatziapostolou A., Zergioti I. (2018). Single Step Laser Transfer and Laser Curing of Ag NanoWires: A Digital Process for the Fabrication of Flexible and Transparent Microelectrodes. Materials.

[38] Lavrenko V.A., Malyshevskaya A.I., Kuznetsova L.I., Litvinenko V.F., Pavlikov V.N. (2006). Features of High-Temperature Oxidation in Air of Silver and Alloy Ag−Cu, and Adsorption of Oxygen on Silver Powder. Metall. Met. Ceram..

[39] Warrier P., Teja A. (2011). Effect of particle size on the thermal conductivity of nanofluids containing metallic nanoparticles. Nanoscale Res. Lett..

