# Supplementary material for: Selective Laser Sintering of Laser Printed Ag Nanoparticle Micropatterns at High Repetition Rates
Source: Materials (Basel). 2018 Oct 31;11(11):2142. doi: 10.3390/ma11112142 (PMC6266122; doi:10.3390/ma11112142)
Supplement: Supplementary file 1 [file materials-11-02142-s001.pdf]

materials

MDPI

Article

Selective Laser Sintering of Laser Printed Ag Nanoparticle Micropatterns at High Repetition Rates

Filimon Zacharatos [1,*], Ioannis Theodorakos [1], Panagiotis Karvounis [2], Simon Tuohy [3], Nuno Braz [3], Semyon Melamed [4], Ayala Kabla [4], Fernando de la Vega [4], Kostas Andritsos [1], Antonios Hatziapostolou [2], Dimitris Karnakis [3] and Ioanna Zergioti [1]

1 Physics Department, Zografou Campus, National Technical University of Athens, Athens, 15780, GR; jtheod@mail.ntua.gr (I.T.); kostas_andritsos13@hotmail.com (K.A.); zergioti@central.ntua.gr (I.Z.)
2 School of Engineering, University of West Attica, Campus 1, Aigaleo, 12243, GR; panos.karvounis12@gmail.com (P.K.); ahatzi@teiath.gr (A.H.)
3 Oxford Lasers Ltd. 8 Moorbrook Park, Didcot, Oxon OX11 7HP, UK; simon.tuohy@oxfordlasers.com (S.T.); nuno.braz@oxfordlasers.com (N.B.); Dimitris.Karnakis@oxfordlasers.com (D.K.)
4 PV Nano Cell Ltd., 8 Hamasger st., P.O. Box 236 Migdal Ha'Emek, 2310102 Israel; simon@pvnanocell.com (S.M.); ayala@pvnanocell.com (A.K.); fernando@pvnanocell.com (F.d.l.V.)
* Correspondence: fzach@mail.ntua.gr; Tel.: +30-210-772-1712

Received: 29 September 2018; Accepted: 25 October 2018; Published: date.

Below two supplementary Figures S1 and S2, can be found.

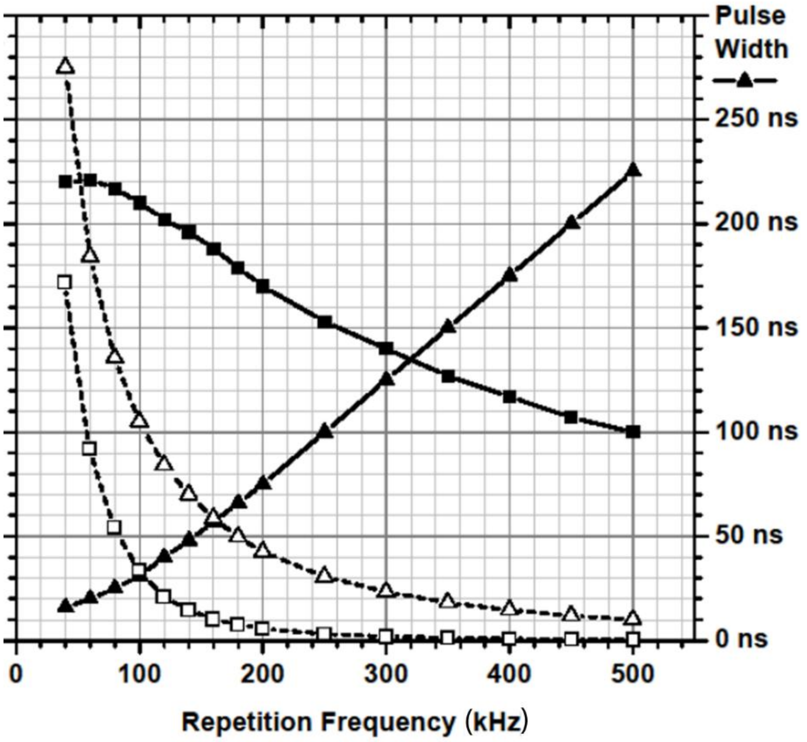

Figure S1. Performance of the high repetition rate laser utilized in this study according to the manufacturer. Pulse width increases for increasing Repetion Frequency.

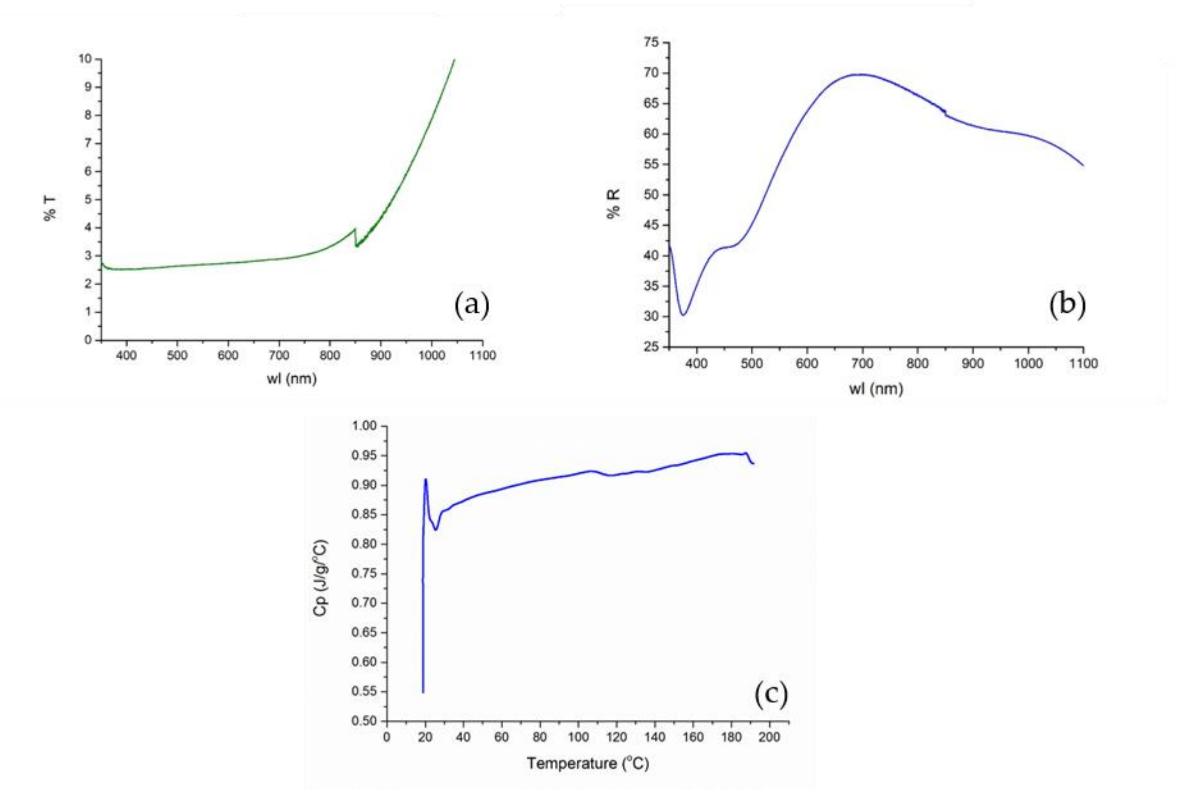

Figure S2. Measured T (**a**); R (**b**) spectra for 180 nm thick spin coated Ag nanoparticle ink for the 350–1100nm range and (**c**) Cp vs T for the same ink.

© 2018 by the authors. Licensee MDPI, Basel, Switzerland. This article is an open access article distributed under the terms and conditions of the Creative Commons Attribution (CC BY) license (http://creativecommons.org/licenses/by/4.0/).
